# Disentangling Sub-Millisecond Processes within an Auditory Transduction Chain

**DOI:** 10.1371/journal.pbio.0030008

**Published:** 2005-01-04

**Authors:** Tim Gollisch, Andreas M. V Herz

**Affiliations:** **1**Institute for Theoretical Biology, Humboldt University, BerlinGermany; **2**Bernstein Center for Computational Neuroscience, BerlinGermany; Harvard UniversityUnited States of America

## Abstract

Every sensation begins with the conversion of a sensory stimulus into the response of a receptor neuron. Typically, this involves a sequence of multiple biophysical processes that cannot all be monitored directly. In this work, we present an approach that is based on analyzing different stimuli that cause the same final output, here defined as the probability of the receptor neuron to fire a single action potential. Comparing such iso-response stimuli within the framework of nonlinear cascade models allows us to extract the characteristics of individual signal-processing steps with a temporal resolution much finer than the trial-to-trial variability of the measured output spike times. Applied to insect auditory receptor cells, the technique reveals the sub-millisecond dynamics of the eardrum vibration and of the electrical potential and yields a quantitative four-step cascade model. The model accounts for the tuning properties of this class of neurons and explains their high temporal resolution under natural stimulation. Owing to its simplicity and generality, the presented method is readily applicable to other nonlinear cascades and a large variety of signal-processing systems.

## Abstract 2

Comparing auditory stimuli that give the same neural response within the framework of a computational model, the authors extract intermediary signal-processing steps with sub- millisecond temporal resolution

## Introduction

**Figure 1:**
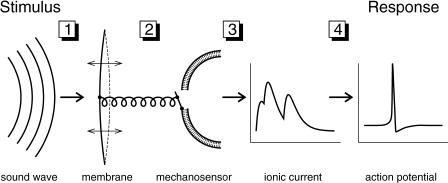
Sequential Processing in the Auditory Transduction Chain A sequence of several steps transforms an incident sound wave into a neural spike response. (1) Mechanical coupling. The acoustic stimulus induces vibrations of a mechanical membrane (basilar or tympanic membrane). (2) Mechanosensory transduction. The deflections cause the opening of mechanosensory ion channels in the membrane of a receptor neuron. Many details of this transduction process are still unknown. The depicted schematic coupling follows the gating-spring model proposed for mechanosensory transduction in hair cells [43]. (3) Electrical integration. The electrical charge due to the transmembrane current accumulates at the cell membrane. (4) Spike generation. Action potentials are triggered by voltage-dependent currents. Each of these four steps transforms the signal in a specific way, which may be nearly linear (as for the eardrum response) or strongly nonlinear (as for spike generation, which is subject to thresholding and saturation). In general, the illustrated steps may contain further sub-processes such as cochlear amplification or synaptic transmission between hair cells and auditory nerve fibers. For the auditory periphery of locusts investigated in the present study, this schematic picture resembles anatomical findings [18], which reveal that the receptor neurons are directly attached to the eardrum and that they send their action potentials down the auditory nerve without any further relay stations.

Animals and human beings rely on accurate information about their external environment and internal state for proper behavioral reactions. This vital requirement has led to a large variety of highly sophisticated sensory systems [1]. A common feature, though, is the step-by-step conversion of the incoming signal through multiple sequential transformations. In auditory systems, for example, air-pressure fluctuations induce oscillations of mechanical resonators such as the eardrums, basilar membranes, and hair sensilla [2,3,4,5]. These oscillations cause the opening of mechanosensory ion channels in auditory receptor cells [6,7,8]. The resulting electrical currents change the cells' membrane potentials. This, in turn, activates voltage-dependent ion channels that eventually trigger action potentials, which are passed to higher brain areas for further information processing (Figure 1). Each processing step induces a transformation of the stimulus representation that may include rectification, saturation, and temporal filtering. In the mammalian ear, this processing sequence is extended by nonlinear mechanical amplification and feedback [9], which influence the individual processing steps. Similar multi-step sequences of biophysical or biochemical transduction processes underlie the proper function of all sensory and many other signaling systems.

We here show that it is possible to extract fine temporal details of individual processes within such signal-processing chains from observing the output activity alone. This progress results from a new method that extends an experimental strategy well known from measuring threshold curves in neurobiology [10] or applying equivalence criteria in psychophysics [11]: varying stimulus parameters such that the investigated pathway, cell, or system stays at a constant level of output activity. The key to the new method is to compare different stimuli within these measured iso-response sets in such a way that single processing steps can be dissociated. A cascade model is used as a mathematical framework to infer the salient features of the individual processes. This allows us to quantitatively characterize the signal-processing dynamics even under in vivo conditions.

Unlike many classical approaches of systems identification, the method is not based on temporal correlations between the input and output; hence, the time resolution of the method is not limited by the output precision of the system under study. In a spike-based analysis of neural response properties, this allows us to assess the dynamical features of the involved processes with considerably higher resolution than suggested by the spike jitter.

A particularly fine temporal resolution is needed to analyze signal processing in auditory systems that solve complex tasks such as sound localization, echolocation, and acoustic communication [12,13,14,15]. Here, even single receptor cells display extraordinary sub-millisecond precision [14,16,17], with the underlying signal-processing steps involving yet shorter time scales. How these individual steps operate over short times and eventually allow such remarkable precision is largely unknown because of the high vulnerability of the auditory periphery. This calls for methods based on neurophysiological measurements from a remote downstream location such as the auditory nerve, so that the mechanical structures of the ear remain intact.

As a suitable model system to study signal processing in the ear, we chose the auditory periphery of the locust (Locusta migratoria). Its anatomy is well characterized [18], and the auditory nerve is easily accessible for electrophysiological recordings. The nerve contains the axons of the receptor cells. These can be roughly divided into two groups according to their frequency of maximum sensitivity, which lies near 5 kHz for low-frequency receptor cells and around 15 kHz for high-frequency receptor cells. The mechanical structure of the locust system is simpler than that of mammals, as the receptor cells are directly attached to the tympanic membrane, the animal's eardrum. Also, in contrast to the signal amplification in the vertebrate cochlea, there are no known feedback loops, a circumstance which facilitates the modeling. General features of mechanoreceptors, on the other hand, are surprisingly similar across species and are also shared by hair cells in the mammalian inner ear [8].

## Results

**Figure 2:**
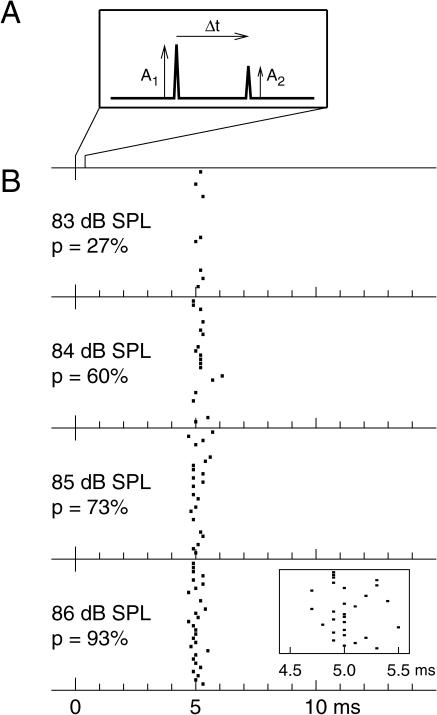
Receptor Neuron Responses for Two-Click Stimuli (A) Stimulus parameters. Acoustic stimuli consisted of two short clicks with amplitudes *A*_1_ and *A*_2_, respectively, separated by a peak-to-peak interval Δ*t*. The clicks were triangular and had a total width of 20 μs. The peak-to-peak interval was generally less than 1.5 ms. (B) Raster plots of spike responses. Spike times obtained from a single receptor neuron with four different peak intensities (83–86 dB SPL) are shown for 30 runs each. For the different intensities, both click amplitudes were varied while their ratio was kept fixed, with intensity values referring to the larger click amplitude. The inter-click interval in this example was 40 μs. The values of *p* denote the measured spike probabilities. The inset displays spike times from the strongest sound stimulus at higher magnification. All spikes fall in a temporal window between 4.5 and 5.5 ms after stimulation. Spike times were recorded with a temporal resolution of 0.1 ms. These data illustrate that the response of the receptor cell is well described by the occurrence probability of a single spike in a rather broad time window, for example, between 3 and 10 ms after stimulus presentation. As is often observed for these receptor cells, there is virtually no spontaneous activity.

To analyze signal processing in the locust ear, we performed intracellular recordings in vivo from single receptor-cell axons in the auditory nerve. The stimuli consisted of two short clicks. The clicks were sound-pressure pulses with peak amplitudes *A*_1_ and *A*_2_, respectively, and were separated by a short time interval, Δ*t* (Figure 2A; see also Figure S1 for microphone recordings). For such stimuli, the receptor cell fired at most one action potential per double click; stimulus intensity hardly influenced spike timing, but strongly affected spike probability, as shown in Figure 2B. The response strength may thus be described by the probability that a spike occurs within a certain time window after the two clicks.

For fixed time interval Δ*t,* an iso-response set consists of those combinations of *A*_1_ and *A*_2_ that lead to the same predefined spike probability *p*. Since the spike probability increases with the click amplitudes, *A*_1_ and *A*_2_ can easily be tuned during an experiment to yield the desired value of *p* (see Materials and Methods). The tuning scheme was applied for stimulus patterns with different relative sizes of the two clicks, so that a multitude of different combinations of *A*_1_ and *A*_2_ corresponding to the same *p* was obtained. Rapid online analysis of the neural responses and automatic feedback to the stimulus generator made it possible to apply this scheme despite the time limitations of the in vivo experiments.

**Figure 3:**
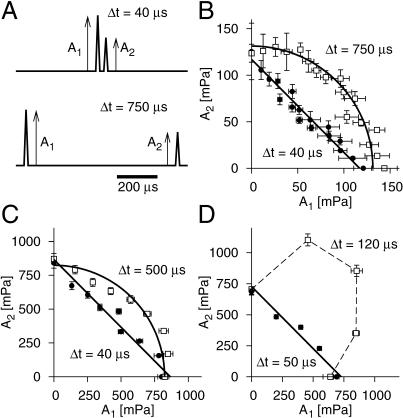
Measurements of Iso-Response Sets and Identification of Relevant Stimulus Parameters (A) Acoustic stimuli. The stimuli consisted of two short clicks with amplitudes *A*_1_ and *A*_2_ that were separated by a peak-to-peak interval Δ*t*, here shown for Δ*t* = 40 μs (upper trace) and Δ*t* = 750 μs (lower trace). (B–D) Examples of iso-response sets from three receptor cells. Here, as throughout the paper, iso-response sets correspond to a spike probability of 70%. Each panel shows iso-response sets from a single receptor cell for two different values of Δ*t,* one smaller than 100 μs (filled circles) and one larger (open squares). The solid lines denote fits to the data of either straight lines or circles. The values for Δ*t* used in the experiments are indicated in the respective panels. All error measures display 95% confidence intervals. For the short intervals, the data are well fitted by straight lines (*A*_1_ + *A*_2_ = constant). For the long intervals in (B) and (C), circles (*A*_1_^2^ + *A*_2_^2^ = constant) yield good fits; a slight asymmetry is clearly visible in (C). The data for the intermediate inter-click interval Δ*t* = 120 μs in (D) are not well fitted by either of these shapes. Here, the measured points are connected by a dashed line for visual guidance. Note that in (B) the overall sensitivity of the neuron seems to have changed; the intersections of the straight line and the circle with the x- and y-axis do not match exactly although the stimulus in these cases is the same, a single click. The reason may be either a slow adaptation process or a slight rundown of the recording over the experimental time of around 30 min. However, this does not account for the more prominent differences in shape of the two iso-response sets. These examples demonstrate that on different time scales, different stimulus parameters are relevant for the transduction process, the amplitude *A* of a sound stimulus for short times and its energy *A* ^2^ for long times.

Figure 3 shows typical examples of such iso-response sets, measured for different time intervals Δ*t*. For each of the three cells displayed, two distinct values of Δ*t* were used. The sets can be used to identify stimulus parameters that govern signal processing at a particular time scale. Most importantly, the iso-response sets exhibit specific shapes that vary systematically with Δ*t*. For short intervals (below approximately 60 μs), the sets generally lie on straight lines, at least for low-frequency receptor cells. High-frequency receptor cells do not display straight lines even at the smallest Δ*t* used in the experiment (40 μs) for reasons that will become apparent later. For long intervals (between approximately 400 and 800 μs, depending on the cell), the iso-response sets fall onto nearly circular curves. Note that in Figure 3C, the iso-response set for Δ*t* = 500 μs deviates from the symmetry between *A*_1_ and *A*_2_. In Figure 3D, the inter-click interval of Δ*t* = 120 μs fell in neither of the two regimes discussed above, and the corresponding iso-response set shows a particularly bulged shape. Recordings from a total of eight cells agree with the observations from the three examples displayed in Figure 3.

The two prominent shapes of the iso-response sets—straight lines and circles—reflect two different processing steps in the auditory transduction chain. A straight line implies that the linear sum, *A*_1_ **+** *A*_2_, of both click amplitudes determines the spike probability and demonstrates that the sound pressure is most likely the relevant stimulus parameter. Such linear summation of the pressure on short time scales is not surprising, considering the mechanical properties of the eardrum; owing to its mechanical inertia, rapidly following stimuli can be expected to superimpose. This interpretation is in agreement with laser-interferometric and stroboscopic observations of the eardrum, which have demonstrated that it reacts approximately linearly to increases in sound pressure [3,19].

For the longer intervals, on the other hand, the iso-response sets are circles to good approximation, indicating that the quadratic sum, or *A*_1_^2^ **+** *A*_2_^2^, now determines the spike probability. It follows that the sound energy, which is proportional to the squared pressure, is the relevant stimulus parameter on this time scale. This quadratic summation represents a fundamentally different way of stimulus integration from that of the linear summation on short time scales and indicates the involvement of a different biophysical process. A process that can mediate stimulus integration over longer intervals is the accumulation of electrical charge at the neural membrane. According to this explanation, the electrical potential induced by a click is proportional to the click's energy; contributions from consecutive clicks are then summed approximately linearly because of the passive membrane properties. This is in accordance with earlier investigations for stationary sound signals that revealed an energy dependence of the neurons' firing rate [20]. We conclude that in between the mechanical vibration of the eardrum and the accumulation of electrical charge at the neural membrane, there is a squaring of the transmitted signal. This squaring may be attributed to the core process of mechanosensory transduction, i.e., the opening of ion channels by the mechanical stimulus.

The above findings motivate the following mathematical model, which describes how a stimulus consisting of two sound clicks is transformed into a spike probability. Within the model, a single click of amplitude *A* generates a vibration of the tympanum with strength *X = c*_1_ *·A,* i.e., linear in the amplitude with a proportionality constant *c*_1_. This mechanical vibration leads to a membrane potential, whose effect on the generation of the spike some time *T* after the click is given by *J = c*_2_·*X* ^2^ *= c*_2_ *·*(*c*_1_ *·A*)^2^, i.e., quadratic in the amplitude with an additional proportionality constant *c*_2_. The square follows from the circular shape of the iso-response sets for longer time scales, which indicated that a quadratic operation must take place before the accumulation of charge at the neural membrane. Finally, the spike probability *p* is given by a yet unknown function *p = g*(*J*). As *J* is the relevant quantity determining spike probability, we also refer to it as “effective stimulus intensity.” The model contains a freedom of scaling; any proportionality constants in *J* can be absorbed into the function *g*(*J*). To simplify the notation, we thus set *c*_1_ = *c*_2_ = 1 and obtain *X = A* for the strength of the mechanical vibration and *J = X* ^2^ *= A* ^2^ for the effective stimulus intensity in response to a single click.

Note that in this picture, the mechanical vibration and the membrane potential are each captured by a single quantity that does not describe the time course of the corresponding processes, but rather their integrated strength in response to a click. In general, the conversion of the mechanical vibration into a membrane potential as well as the spike generation are dynamical processes that do not happen at a single moment in time. For simplicity, however, one may think of *X* as describing the velocity of the mechanical vibration immediately after the click and *J* as capturing the membrane potential at the time of spike generation.

For the two-click stimulus with amplitudes *A*_1_ and *A*_2_, respectively, we choose the first click to be small enough so that it does not lead to a spike by itself. The measured action potential is thus elicited at some time *T* after the second click. To derive the model equation for this experimental situation, we divide the time from the first click to spike generation into the period between the two clicks and the period following the second click.

Let us start by focusing on the inter-click interval. After the first click, the mechanical vibration has the strength *X*_1_ = *A*_1_. However, how much electrical charge accumulates during the inter-click interval to influence spike generation at time *T* after the second click depends on the length Δ*t* of the inter-click interval. This effect is incorporated by a Δ*t*-dependent scaling factor *Q*(Δ*t*) into the model and results in a first contribution from the first click to spike generation given by *J*_1_ *= A*_1_^2^ *·Q*(Δ*t*). Since *Q*(Δ*t*) denotes the effect of the first click within the inter-click interval only, it should vanish in the limit of very small Δ*t*.

Let us now consider the remaining time before spike generation. After the second click, the mechanical vibration is due to a superposition of both clicks. For short inter-click intervals, the straight iso-response lines suggest a simple addition of the two click amplitudes; in general, however, the contribution of the first click to the membrane vibration after the second click will again depend on the inter-click interval Δ*t*. This is modeled by a scaling factor *L*(Δ*t*), i.e., the vibration after the second click has a strength *X*_2_ = *A*_1_· *L*(Δ*t*) + *A*_2_. Accordingly, the effect of the two-click vibration on the membrane potential at time *T* after the second click is *J*_2_ *=* (*X*_2_)^2^ *=* (*A*_1_ *· L*(Δ*t*) + *A*_2_)^2^. For very small Δ*t, L*(Δ*t*) should approach unity to account for the equal contribution of both clicks for vanishing inter-click intervals. The total effective stimulus intensity is then given by



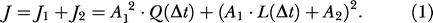



This quantity determines the spike probability *p* via the relation *p* = *g*(*J*).

How does this model explain the particular shapes of the iso-response sets in Figure 3? The linear and the circular iso-response sets apparently correspond to the two special cases: (1) *L*(Δ*t*) = 1 and *Q*(Δ*t*) = 0 (straight line) and (2) *L*(Δ*t*) = 0 and *Q*(Δ*t*) = 1 (circle).

We can therefore regard equation 1 as a minimal model incorporating linear as well as quadratic summation, as suggested by the measured iso-response sets. Based on the experimental data, we expect that the first case is approximately fulfilled for small Δ*t* and the second case in some range of larger Δ*t*. In our biophysical interpretation, the first case means that the two clicks are added at the tympanic membrane (*L*(Δ*t*) ≈ 1), but the short interval between the two clicks prevents a substantial accumulation of charge from the first click alone (*Q*(Δ*t*) ≈ 0), as already discussed above. The second case may be found for Δ*t* long enough that the mechanical vibration has already decayed (*L*(Δ*t*) ≈ 0). The two clicks are then individually squared, i.e., they independently lead to two transduction currents. The currents add up if the time constant of the neural membrane is significantly longer than the inter-click interval (*Q*(Δ*t*) ≈ 1).

In the two limiting cases, equation 1 is symmetric with respect to *A*_1_ and *A*_2_, reflecting the symmetry of, e.g., the data in Figure 3B. However, for values of Δ*t* where neither of the two cases is strictly fulfilled, this symmetry of the iso-response sets will be distorted, as is noticable for the longer Δ*t* in Figure 3C. Other sets of values for *L*(Δ*t*) and *Q*(Δ*t*) may lead to very different iso-response shapes, as in Figure 3D.

Equation 1 presents a self-contained model for click stimuli and is sufficient to analyze the temporal characteristics of the individual steps. It can be interpreted as a signal-processing cascade that contains two summation processes, one linear in the click amplitudes and one quadratic. For click stimuli, the functions *L*(Δ*t*) and *Q*(Δ*t*) are thus filter functions associated with the linear and quadratic summation, respectively.

Despite the simple structure of the model, the filters *L*(Δ*t*) and *Q*(Δ*t*) can be expected to retain the salient features of the underlying biophysical processes such as frequency content and integration time. In Protocol S1, we show that equation 1 can be obtained in an a posteriori calculation from a generalized cascade model and that this derivation leads to an interpretation of *L*(Δ*t*) as the velocity of the mechanical vibration and of *Q*(Δ*t*), at least for large enough Δ*t,* as the time course of the membrane potential following a click. In this generalized model, the input signal is an arbitrary sound pressure wave *A*(*t*), and the effective stimulus intensity is a continuous function of time, *J*(*t*), which is given by



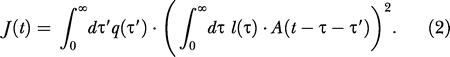



**Figure 4:**
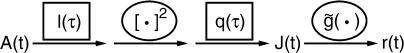
Generalized Cascade Model of the Auditory Transduction Chain The model is composed of a sequence containing two linear temporal filters, *l*(*τ*) and *q*(*t*), and two static nonlinear transformations, namely a quadratic nonlinearity and an output nonlinearity *g˜*(·), which may differ from the nonlinearity *g*(·) of the click-stimulus model (see Protocol S1). First, the stimulus *A*(*t*) is convolved with the filter *l*(*τ*) (linear integration). Second, the result is squared (nonlinear transformation). Third, the result of the previous step is convolved with the filter *q*(*τ*), yielding the effective stimulus intensity *J*(*t*) (linear integration). Fourth, a final transformation *g˜* of *J*(*t*) (nonlinear transformation) determines the response, which in this generalized model is the time-dependent firing rate *r*(*t*). The model thus corresponds to an LNLN cascade. This abstract structure directly follows the sequential configuration of the biophysical processing steps shown in Figure 1.

Here, the input *A*(*t*) is first convolved with a temporal filter, *l*(*τ*), the result is squared and subsequently convolved with a second filter, *q*(*τ*), as depicted in Figure 4. The filters *l*(*τ*) and *q*(*τ*) have characteristics similar to the click-version filters *L*(Δ*t*) and *Q*(Δ*t*), but are not identical to them. Their relations follow from the calculation in Protocol S1. As we here focus on click stimuli, we will use the simpler equation 1 to evaluate the temporal structures of *L*(Δ*t*) and *Q*(Δ*t*).

Note that we interpret equation 1 to yield the spike probability after the second click. If the first click is large and the second small, however, the first click alone may account for some of the observed spikes; clearly this is the case when the second click vanishes. This is not captured by equation 1, and one might expect that, for large values of *A*_1_, these additional spikes lead to measured values of *A*_2_ that are slightly smaller than expected for a circular iso-response set. The data in Figure 3, however, suggest that this effect is small and not picked up by our experiment. Nevertheless, for the following quantitative study, we will keep the first click always on a level where the click by itself does not contribute substantially to the spike probability.

**Figure 5:**
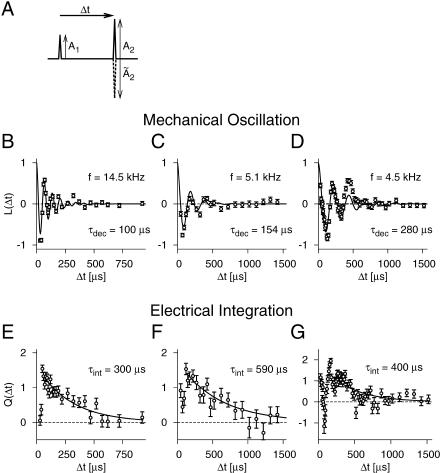
Temporal Structure of the Mechanical Oscillation and Electrical Integration (A) Stimulus patterns. Two clicks were presented, separated by a time interval Δ*t*. The first click (amplitude *A*_1_) was held constant throughout this experiment. The second click was presented in the same direction as the first click (solid line, amplitude *A*_2_) or in the opposite (“negative”) direction (dashed line, amplitude *Ã*_2_). The click amplitudes *A*_2_ and *Ã*_2_ were adjusted to fall in the desired iso-response set. (B–G) Mechanical oscillation and electrical integration of a high-frequency (B and E) and two low-frequency (C and F, and D and G, respectively) receptor neurons. (B–D) Time course of the eardrum vibration. The individual values (circles) were calculated from the measured values of *A*_2_ and *Ã*_2_ for each Δ*t*. The results are compared with a theoretical curve from a damped harmonic oscillator (solid line) with fundamental frequency *f* and decay time constant *τ*_dec_ fitted to the data. (E–G) Time course of the electrical integration process. The measured data are compared to an exponential fit (solid line) with a time constant *τ*_int_.

The previous experiment showed that the separate effects of the two summation processes can be discerned for short and long time intervals. For intermediate Δ*t,* however, their dynamics may largely overlap. Is it nevertheless possible to design an experiment that directly reveals the whole time course of the mechanical vibration *L*(Δ*t*) and the electrical integration *Q*(Δ*t*)? This would provide a parameter-free description of both processes and advance the quantitative understanding of the auditory transduction dynamics. To reach this goal, we again measure iso-response sets. As before, we exploit that for fixed Δ*t,* any pair of click amplitudes (*B*_1_, *B*_2_) should result in the same spike probability *p* as the pair (*A*_1_, *A*_2_) as soon as *J*(*A*_1_, *A*_2_) = *J*(*B*_1_, *B*_2_). It is this straightforward relation that allows us to determine both *L*(Δ*t*) and *Q*(Δ*t*) independently of each other. In fact, some appropriate set of measurements that fulfill the iso-response relation is all that is needed to calculate *L*(Δ*t*) and *Q*(Δ*t*). Illustrating this concept, we now proceed with a particularly suited choice of stimulus patterns, which keeps the mathematical requirements for the calculation at a minimum. For each Δ*t,* we measure two different iso-response stimuli, and as a key feature, one of these has a “negative” second click, i.e., a sound-pressure pulse pointing in the opposite direction as the first click, as depicted in Figure 5A. Mathematically, this choice of stimulus patterns leads to two simple equations for the two unknowns *L*(Δ*t*) and *Q*(Δ*t*), which can be solved explicitly, as explained in Materials and Methods. By repeating such double measurements for different values of Δ*t,* the whole time course of *L*(Δ*t*) and *Q*(Δ*t*) is obtained.

**Figure 6:**
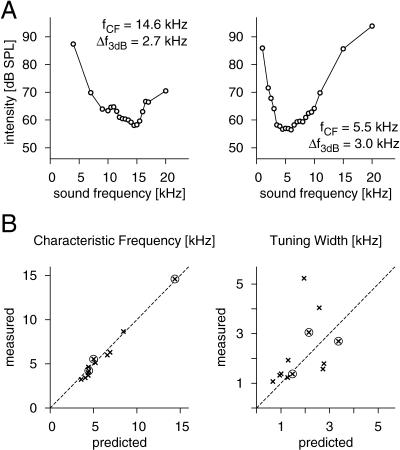
Predictions of Tuning Characteristics (A) Tuning curves for the same two cells as in Figure 5B and 5E, and 5C and 5F, respectively. The data show the intensity required to drive a receptor cell at a firing rate of 150 Hz for different sound frequencies in the range of 1 to 40 kHz. The characteristic frequency *f*_CF_ is determined as the minimum of the tuning curve, and the tuning width Δ*f*_3dB_ as the width of the curve 3 dB above the minimum value. (B) Comparison of the predicted and measured characteristic frequency and the tuning width. The predictions were obtained from the fundamental frequency and decay time constant of the measured filter *L*(Δ*t*); the measured values are taken from the tuning curves as in (A) (*n* = 12). The encircled data points correspond to the three examples shown in Figure 5. The width of the tuning curves is notoriously difficult to assess quantitatively, as it depends sensitively on an accurate determination of the intensity minimum of the tuning curve. This contributes strongly to the differences of the tuning-width values.

Figure 5 shows examples of *L*(Δ*t*) and *Q*(Δ*t*) for three different cells. *L*(Δ*t*) displays strong oscillatory components, as was observed for all cells. This property presumably reflects the eardrum's oscillation at the attachment site of the receptor cell. The detailed temporal structure of *L*(Δ*t*) now allows us to investigate the salient features of this oscillation. To quantify our findings, we fit a damped harmonic oscillation to the measured data for *L*(Δ*t*) and extract the fundamental frequency as well as the decay time constant. We can use these values to predict the neuron's characteristic frequency (the frequency of highest sensitivity) and the width of its frequency-tuning curve. Figure 6 shows the comparison of these predictions with traditional measurements of the tuning curves for all 12 cells measured under this experimental paradigm with sufficient sampling to extract *L*(Δ*t*). The remarkable agreement confirms that the new analysis faithfully extracts the relevant, cell-specific properties of the transduction sequence. The correspondence between the tuning characteristics and the filter *L*(Δ*t*) also explains why high-frequency receptor cells do not feature straight lines for their iso-response sets even at the shortest inter-click interval (40 μs) used in the experiment. For those cells, *L*(Δ*t*) decays rapidly, thus not allowing access to the region where *L*(Δ*t*) ≈ 1.

The short initial rise phase of the measured *Q*(Δ*t*) in Figure 5E and 5F illustrates the rapid buildup of the membrane potential after a click. The exponential decay following this phase suggests that the accumulated electrical charge decays over time owing to a leak conductance. Previously, the time constant could not be measured because of difficulties in obtaining recordings from the somata or dendrites of the auditory receptor cells. Using our new method, we find time constants in the range of 200 to 800 μs. These values are small compared to time constants in more central parts of the nervous system, reflect the high demand for temporal resolution in the auditory periphery, and explain the high coding efficiency of the investigated receptor neurons under natural stimulation [21].

In most of our recordings, the temporal extent of the filter *L*(Δ*t*) was considerably smaller than that of *Q*(Δ*t*). This usually leads to a region around a Δ*t* of 400–800 μs, depending on the specific cell, where *L*(Δ*t*) ≈ 0 and *Q*(Δ*t*) is still near unity. These findings correspond to the circular iso-response sets of the initial experiment.

Towards very small Δ*t,* on the other hand, the data show that *Q*(Δ*t*) usually decreases strongly. As explained earlier, this is expected from the linear iso-response sets, and it is observed exemplarily in the data shown in Figure 5E and 5F. In addition, the first few 100 μs of the data may show considerable fluctuations of *Q*(Δ*t*) for some recordings, as in Figure 5G. Different effects may influence this early phase of *Q*(Δ*t*). (1) The electrical potential might be shaped by further dynamics in addition to the low-pass properties of the neural membrane, such as inactivation of the transduction channels or electrical resonances as found in some hair cells [6]. (2) The fluctuations could reflect the oscillatory influx of current following from the oscillation of the eardrum. In other words, the low-pass filtering of the neural membrane may not be strong enough to quench all oscillatory components of the transduction currents. The resulting effect on the filter *Q*(Δ*t*)—though too small to be picked up reliably by the present experiments—can be observed in simulations of the processing cascade, see Figure S2. At present, we cannot distinguish between these two interpretations. More detailed future experiments, however, may allow a quantitative test of these hypotheses.

**Figure 7:**
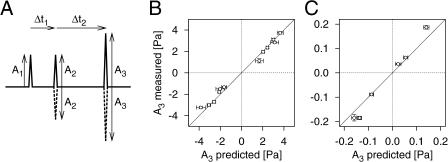
Model Predictions for Three-Click Stimuli (A) Stimulus patterns. The stimuli consisted of three clicks with amplitudes *A*_1_, *A*_2_, and *A*_3_ that were separated by time intervals Δ*t*_1_ and Δ*t*_2_, respectively. The second and third clicks were either given in the same or opposite (“negative”) direction as the first click. *A*_1_ and *A*_2_ were set equal and held constant, and *A*_3_ was adjusted to yield a spike probability of 70%. The following pairs of time intervals (Δ*t*_1_, Δ*t*_2_) were applied: (100 μs, 100 μs), (100 μs, 200 μs), and (200 μs, 100 μs). (B and C) Predicted and measured amplitudes of the third click for two different cells. Predictions were made after *L*(Δ*t*) and *Q*(Δ*t*) had been measured with two-click experiments such as in Figure 5. The comparison between predicted and measured values for *A*_3_ therefore contains no free parameters. The model equation for three-click stimuli is presented in Materials and Methods. As demonstrated by these data, the model allows quantitatively accurate predictions.

Measuring the mechanical and electrical response dynamics, *L*(Δ*t*) and *Q*(Δ*t*), completes the model. In order to test its validity and suitability to make quantitative predictions, we investigated the model's performance on a different class of stimuli, namely combinations of three short clicks. Having measured the required values for *L*(Δ*t*) and *Q*(Δ*t*) with two-click stimuli as in the previous experiment (see Figure 5), we now ask the following question: if we keep the first two clicks small enough that they do not lead to a spike response, can we predict the size of the third click required to reach a given spike probability? We can use the measured values of *L*(Δ*t*) and *Q*(Δ*t*) to calculate these predictions and experimentally test them by performing a series of three-click iso-response measurements. This experiment was performed on three different cells; one cell featured an unusually high response variability, and results from the other two cells are shown in Figure 7. The agreement between the predicted and the true click amplitudes shows that the model yields quantitatively accurate results.

## Discussion

We have presented a novel technique to disambiguate single processing steps within a larger sensory transduction sequence and to analyze their detailed temporal structures. Our approach is based on measuring particular iso-response sets, i.e., sets of stimuli that yield the same final output, and on specific quantitative comparisons of such stimuli to dissociate the individual processes. For the investigated auditory transduction chain in the locust ear, this strategy led to a precise characterization of two consecutive temporal integration processes, which we interpret as the mechanical resonance of the eardrum and the electrical integration of the attached receptor neuron. The method revealed new details of these processes with a resolution far below 1 ms. The results for the time course of the mechanical resonance agree with traditional measurements of tuning curves and show the decay of the oscillation with a temporal precision much higher than expected from the jitter of the measured output signal, the spikes. The time constants of the electrical integration that were extracted from the data had not been accessible by other means.

The analysis resulted in a four-step model of auditory transduction in locusts. The model comprises a series of two linear filters and two nonlinear transformations. The quadratic nonlinearity that separates the two linear filters suggests that the mechanosensory transduction can be described by an energy-integration mechanism, as the squared amplitude corresponds to the oscillation energy of the tympanum. This quadratic form was derived from the circular shape of the iso-response sets for longer time scales Δ*t* and is in accordance with the energy-integration model that was found to capture the sound-intensity encoding of stationary sound signals in these cells [20]. Furthermore, the direct current component of the membrane potential in hair cells is also proportional to sound energy [22], and in psychoacoustic experiments, energy integration accounts for hearing thresholds [23,24,25,26]. However, a recent analysis of response latencies in auditory nerve fibers and auditory cortex neurons in cats suggests an integration of the pressure envelope for determining thresholds [27]. This effect may be attributable to the synapse between the hair cell and the auditory nerve fiber in the mammalian ear. In the locust ear, this synapse does not exist, as the fibers are formed by the axons of the receptor neurons themselves.

Although the quadratic nonlinearity is fully consistent with our data, there is a second possibility within the general cascade model, equation 2, namely, squaring after rectification. From a biophysical point of view, this would be expected if the mechanosensory ion channels can only open in one direction. Based on the current data, we cannot distinguish between these two possibilities. As the two scenarios should lead to slightly different response characteristics, future high-resolution experiments should be able to resolve this question.

The linear filters *L*(Δ*t*) and *Q*(Δ*t*) were interpreted as the mechanical oscillation of the tympanum and the electrical integration at the neural membrane. Their oscillatory and exponential decay characteristics, respectively, support this view. In principle, however, other processes may well contribute to these characteristics, e.g., electrical resonances as seen in hair cells of the turtle and bullfrog [6,28]. These electrical amplification processes would be expected to influence the filter *Q*(Δ*t*), but our data generally provide little evidence for such effects. Deviations from the exponential decay characteristics in *Q*(Δ*t*) may in part be attributable to the oscillatory influx of charge resulting from the tympanic vibration. This may lead to a small oscillatory component in the early phase of the filter (cf. Protocol S1; Figure S2).

The mechanical coupling in the first step of our model is linear. This is in accordance with mechanical investigations of the tympanum using laser interferometry [3] and stroboscopic measurements [19]. As the short clicks used in our study produce reliable spiking responses only at high sound pressure, however, we cannot exclude the influence of nonlinear coupling at low sound pressure, which has been hypothesized on the basis of distortion-product otoacoustic emissions [29]. In addition, the mechanical properties of the tympanum seem to change slightly under prolonged stimulation and give rise to mechanical adaptation effects with time scales in the 100-ms range [30]. Spike-frequency adaptation also adds a nontrivial feedback term to the minimal feedforward model of Figure 4. Similarly, specific potassium currents and sodium-current inactivation induced by sub-threshold membrane potential fluctuations may complicate the transduction dynamics for more general inputs, but do not leave a signature in the present click-stimulus data.

The model was quantitatively investigated by using combinations of short clicks. The particular structure of these stimuli allowed a fairly simple mathematical treatment. The derivation of equation 1 relied on capturing the mechanical vibration and the membrane potential, respectively, by single quantities in each time period following a click. This was possible because of the expected stereotypic evolution of the dynamic variables during the “silent phases” between and after the clicks. A generalization to arbitrary acoustic stimuli would require a more elaborate model in the form of equation 2 as well as extensions that account for neural refractoriness and adaptation.

Besides its applicability under in vivo conditions, the presented framework has several advantageous properties. First, the method effectively decouples temporal resolution on the input side from temporal precision on the output side by focusing on spike probabilities. In all our measurements, for example, spike latencies varied by about 1 ms within a single recording set owing to cell-intrinsic noise (see Figure 2). Still, we were able to probe the system with a resolution down to a few microseconds. This would not have been possible using classical techniques such as poststimulus time histograms, reverse correlation, and Wiener-series analysis. All these methods are intrinsically limited by the width of spike-time jitter and thus cannot capture the fine temporal details of rapid transduction processes. With our method, the resolution is limited only by the precision with which the sensory input can be applied. For the investigated system, the achievable temporal resolution thus increases by at least two orders of magnitude.

Second, the method is robust against moderate levels of spontaneous output activity, as this affects all stimuli within one iso-response set in the same way. Methods that require measurements at different response levels, on the other hand, are likely to be systematically affected because the same internal noise level may have a different influence at different levels of output activity.

Finally, in many input–output systems, the last stage of processing can be described by a monotonic nonlinearity. Here, this is the relation between the effective stimulus intensity *J* and the spike probability *p* = *g*(*J*), which includes thresholding and saturation. By always comparing stimuli that yield the same output activity, our analysis is independent of the actual shape of *g*(*J*). Preceding integration steps may thus be analyzed without any need to model *g*(*J*). This feature is independent of the specific output measure and applies to spike probabilities, firing rates, or any other continuous output variable.

Let us also note that the method does not require that the time scales of the individual processes be well separated. For the studied receptor cells, mechanical damping was on average about two times faster than electrical integration, and even for cells with almost identical time constants, iso-response measurements led to high-quality data and reliable parameter fits. Nor is the method limited to particularly simple nonlinearities. All that is needed are solid assessments of the iso-response sets. Mathematically, it is straightforward to substitute some or all of the analytical treatments of this work by numerical approaches, if required by the complexity of the identified signal-processing steps. This extension allows one to use a general parametrization of the full processing chain when the nonlinear transformation cannot be estimated from iso-response sets at large and small Δ*t*. Instead, performing more than the two measurements at each intermediate Δ*t* in the second experiment (see Figure 5) will provide additional information that can be exploited to improve the numerical estimates of the nonlinearity.

As in many other approaches of nonlinear systems identification, the development of a quantitative model relies on the prior determination of the appropriate cascade structure. Unfortunately, there is no universal technique for doing so. In many cases, intuition is required to find suitable models, which should eventually be tested by their predictive power. In the present case, the findings of characteristic shapes of the iso-response sets gave a clear signature of two distinct linear filters with a sandwiched quadratic nonlinearity. In addition, this structure was supported by its amenability to straightforward biophysical interpretation. Generalizing our results, specific iso-response sets may aid structure identification in conjunction with a priori anatomical and physiological knowledge. Once the cascade structure is established, the individual constituents can be quantitatively evaluated by specific comparisons of iso-response stimuli. Comparing responses to clicks in positive and negative directions as in this study is in essence similar to the approach used by Gold and Pumphrey [31], who evaluated the perceptual difference between short sine tones with coherent phase relations and sine tones that contained phase-inverted parts in order to estimate the temporal extent of the cochlear filters.

A yet open problem is the inclusion of feedback components. The present approach relies on the feedforward nature of the system to disentangle the individual processing steps. In particular cases, however, the iso-response approach may also aid in separating feedforward and feedback contributions, namely, when the feedback depends purely on the last stage of the processing cascade [30]. In this situation, iso-response measurements lead to a constant feedback contribution, and the analysis of the feedforward components may be carried out as in the present case. The experiment may then be repeated for different output levels to map out the feedback characteristics.

The feedforward model that we have proposed here for the auditory transduction chain has the form of an LNLN (where “L” stands for linear and “N” stands for nonlinear) cascade, composed of two linear temporal integrations and two nonlinear static transformations [32]. Similar signal-processing sequences combining linear filters and nonlinear transformations are ubiquitous at all levels of biological organization, from molecular pathways for gene regulation to large-scale relay structures in sensory systems. In neuroscience, applications range from the sensory periphery, including frog hair cells [33], insect tactile neurons [34], and the mammalian retina [35,36,37], over complex cells in visual cortex [38,39], to psychophysics [40]. These studies are restricted to models that contain a single nonlinear transformation, corresponding to NL, LN, or LNL cascades [32,41]. An extension of these analyses was presented by French et al. [42], who derived an NLN cascade for fly photoreceptors.

Complementary to the correlation techniques underlying the parameter estimations in those models, the method presented in this work provides a new way of quantitatively evaluating and testing cascade models. The increased complexity of the LNLN cascade identified in the present case was made accessible by invoking particular iso-response measurements, and a higher temporal resolution was achieved by focusing on how spike probabilities depend on the temporal stimulus structure instead of relying on temporal correlations between stimulus and response.

Our experimental technique will be most easily applicable to systems whose signal processing resembles the cascade structure investigated here. The general concept of combining different measurements from within one iso-response set covers, however, a much larger range of systems. With increasingly available high-speed computer power for online analysis and stimulus generation, this framework therefore seems well suited to solve challenging process-identification tasks in many signal-processing systems.

## Materials and Methods

### 

#### Electrophysiology

We performed intracellular recordings from axons of receptor neurons in the auditory nerve of adult Locusta migratoria. Details of the preparation, stimulus presentation, and data acquisition are described elsewhere [20]. In short, the animal was waxed to a Peltier element; head, legs, wings, and intestines were removed, and the auditory nerves, which are located in the first abdominal segment, were exposed. Recordings were obtained with standard glass microelectrodes (borosilicate, GC100F-10, Harvard Apparatus, Edenbridge, United Kingdom) filled with 1 mol/l KCl, and acoustic stimuli were delivered by loudspeakers (Esotec D-260, Dynaudio, Skanderborg, Denmark, on a DCA 450 amplifier, Denon Electronic, Ratingen, Germany) ipsilateral to the recorded auditory nerve. The reliability of the sound signals used in this study was tested by playing samples of the stimuli while recording the sound at the animal's location with a high-precision microphone (40AC, G.R.A.S. Sound and Vibration, Vedbæk, Denmark, on a 2690 conditioning amplifier, Brüel and Kjær, Langen, Germany). See Figure S1 for example recordings.

Spikes were detected online from the recorded voltage trace with the custom-made Online Electrophysiology Laboratory software and used for online calculation of spike probabilities and automatic tuning of the sound intensities. The measurement resolution of the timing of spikes was 0.1 ms. During the experiments, the animals were kept at a constant temperature of 30 °C by heating the Peltier element. The experimental protocol complied with German law governing animal care.

#### Measurement of iso-response sets

Since the spike probability *p* of the studied receptor neurons increases monotonically with stimulus intensity, parameters of iso-response stimuli corresponding to the same value of *p* can be obtained by a simple online algorithm that tunes the absolute stimulus intensity. For fast and reliable data acquisition, we chose *p* = 70%. The response latency of the neurons varied by 1–2 ms, so that spike probabilities could be assessed by counting spikes over repeated stimulus presentations in a temporal window from 3 to 10 ms after the first click.

In the first set of experiments, stimulus patterns were defined by fixed ratios of *A*_1_ and *A*_2_, and the tuning was achieved by adjusting the two amplitudes simultaneously. The ratios were chosen so that the angles *α* in the *A*_1_–*A*_2_ plane given by tan*α* = *A*_2_/*A*_1_ were equally spaced. In the second set of experiments, *A*_1_ was kept fixed, and only *A*_2_ was adjusted; similarly, in the three-click experiments, only *A*_3_ was adjusted. In the following, the intensity *I* always refers to the peak amplitude *A*_max_ of the stimulus pattern, measured in decibel sound pressure level (dB SPL),



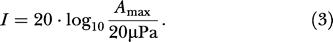



For each stimulus, the absolute intensity *I*_70_ corresponding to a spike probability of 70% was determined online in the following way. Beginning with a value of 50 dB SPL, the intensity was raised or lowered in steps of 10 dB, depending on whether the previous intensity gave a spike probability lower or higher than 70% from five stimulus repetitions. This was continued until rough upper and lower bounds for *I*_70_ were found. From these, a first estimate of *I*_70_ was obtained by linear interpolation. Seven intensity values in steps of 1 dB from 3 dB below to 3 dB above this first estimate were then repeated 15 times. From the measured spike probabilities, a refined estimate of *I*_70_ was obtained by linear regression. Nine intensities from 4 dB above to 4 dB below this value were repeated 30 times (in some experiments 40 times). The final estimate of *I*_70_ was determined offline from fitting a sigmoidal function of the form



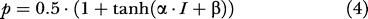



with parameters *α* and *β* to these nine intensity-probability pairs. This relation between *p* and *I* was then inverted to find the intensity and thus the absolute values of the amplitudes that correspond to *p =* 0.7.

#### Extraction of *L*(Δ*t*) and *Q*(Δ*t*) from iso-response sets

The response functions *L*(Δ*t*) and *Q*(Δ*t*) can be obtained independently of each other by combining the results from different measurements within one iso-response set. Here, we derive explicit expressions based on a specific choice of stimuli that are particularly suited for our system. Two measurements are needed to obtain both *L*(Δ*t*) and *Q*(Δ*t*) for given time interval Δ*t*. Each stimulus consists of two clicks. The first click has a fixed amplitude *A*_1_; the amplitude *A*_2_ of the second click at time Δ*t* later is adjusted so that a predefined spike probability *p* is reached. For the second measurement, the experiment is then repeated with a “negative” second click, i.e., a click with an air-pressure peak in the opposite direction from the first click. The absolute value of this click amplitude is denoted by *Ã*_2_. We thus find the two pairs (*A*_1,_ *A*_2_) and (*A*_1_, *Ã*_2_) as elements of an iso-response set. Since the spike probability increases with the effective stimulus intensity *J,* equal spike probability *p* implies equal *J*. The two pairs (*A*_1,_ *A*_2_) and (*A*_1_, *Ã*_2_) therefore correspond to the same value of *J*. According to the model, equation 1, the click amplitudes thus satisfy the two equations



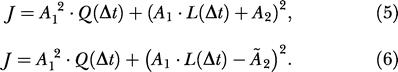



Setting the two right sides equal to each other, we obtain



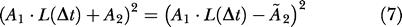



or



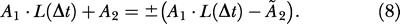



The first solution of this mathematical equation, *Ã*_2_ = − *A*_2_, does not correspond to a physical situation as both *A*_2_ and *Ã*_2_ denote absolute values and are therefore positive. The remaining, second solution reads



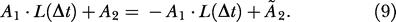



Solving for *L*(Δ*t*), we obtain



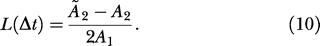



Substituting *L*(Δ*t*) from equation 10 in equation 5 or equation 6, we find



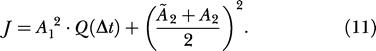



This yields



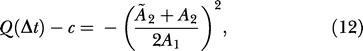



with *c = J*/*A*_1_^2^. As we keep *A*_1_ and *J* constant throughout the experiment, this determines *Q*(Δ*t*) up to the constant *c*. It can be inferred from an independent measurement with a single click: by setting *A*_1_ = 0 in equation 5, we see that *J* corresponds to the square of the single-click amplitude that yields the desired spike probability. Alternatively, *c* can be estimated from the saturation level of *Q*(Δ*t*) for large Δ*t,* as was done in the present study.

The specific form of the effective stimulus intensity, equation 1, led to particularly simple expressions for the response functions *L*(Δ*t*) and *Q*(Δ*t*); see equation 10 and equation 12, respectively. Other nonlinearities may result in more elaborate expressions or implicit equations, but this technical complication does not limit the scope of the presented approach.

#### Data fitting

The datasets for *L*(Δ*t*) were fitted with velocity response functions of a damped harmonic oscillator



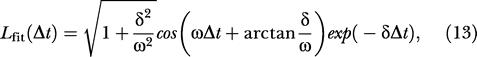



where *ω* and *δ* were optimized for minimizing the total squared error. From these, the fundamental frequency *f* and the decay time constant *τ*_dec_ were determined as *f = ω*/(*2π*) and *τ*_dec_ = 1/*δ*. A simpler fit function of the form



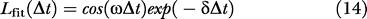



led to essentially indistinguishable results for *f* and *τ*_dec_.

The resonance frequency, which corresponds to the characteristic frequency, *f*_CF_, of the tuning curve, and the tuning width, Δ*f*_3dB_, can be predicted from the fitted values of *ω* and *δ* according to the theory of harmonic oscillators:



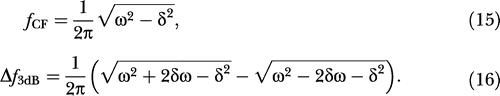



The datasets for *Q*(Δ*t*) were fitted with an exponential decay



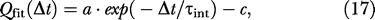



where the parameters *a, τ*_int_, and *c* were adjusted. Here, only data points for Δ*t* > 150 μs were taken into account, as *Q*(Δ*t*) initially shows a rising phase. The obtained value for *c* was used to determine the constant *J*/*A*_1_^2^ in equation 12.

For comparing these predicted values with measurements, the minimum and width of the tuning curves (see Figure 6A) were determined by fitting a quadratic function to the five data points closest to the data point with smallest intensity.

#### Model predictions for three-click stimuli

For stimuli consisting of three clicks with amplitudes *A*_1_, *A*_2_, and *A*_3_ that are separated by time intervals Δ*t*_1_ and Δ*t*_2_, respectively (see Figure 7A), an approximate equation for the effective stimulus intensity *J* can be derived in the following way: The first click induces a tympanic vibration proportional to *A*_1_ and a membrane potential proportional to *A*_1_^2^. Following the second click, the tympanic deflection has become *A*_1_· *L*(Δ*t*_1_) and is augmented by *A*_2_. This yields a membrane potential proportional to (*A*_1_·*L*(Δ*t*_1_) + *A*_2_)^2^. After the third click, the tympanic deflection has evolved to *A*_1_·*L*(Δ*t*_1_ + Δ*t*_2_) + *A*_2_·*L*(Δ*t*_2_) so that the membrane potential is increased by (*A*_1_·*L*(Δ*t*_1_ + Δ*t*_2_) + *A*_2_·*L*(Δ*t*_2_) + *A*_3_)^2^. Summing up the different contributions and approximating the influence of the inter-click intervals on the membrane potential by appropriate factors of *Q,* we find for the effective stimulus intensity



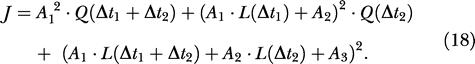



The value of *J* for a predefined spike probability can be measured from a single-click experiment by setting *A*_1_ *= A*_2_ = 0 and tuning *A*_3_ until the desired spike probability is reached. After having measured *L*(Δ*t*) and *Q*(Δ*t*) from two-click experiments, the above equation can be used to predict the amplitude *A*_3_ needed to reach this predefined spike probability for any combination of *A*_1_, *A*_2_, Δ*t*_1_, and Δ*t*_2_.

## Supporting Information

Protocol S1 General Cascade Model (50 KB PDF).

Figure S1 Examples of Click Stimuli The four panels show different examples of stimuli used in our study. Each panel illustrates the computer-generated pulse signal that drives the loud speaker (upper trace) and the resulting air-pressure fluctuations as measured with a high-precision microphone at the site of the animal's ear (lower trace). The computer-generated clicks are triangular with a total width of 20 μs. The stimuli shown are (A) a single click, (B) a double click with a peak-to-peak interval Δ*t =* 50 μs, (C) a double click with Δ*t* = 500 μs, and (D) another double click with Δ*t* = 500 μs whose second click points in the oppositve (“negative”) direction. The measurements of air-pressure fluctuations indicate a slight broadening of the click width and some residual vibrations, but they nevertheless present a good approximation of the sharp original pulses.
(10 KB PDF).

Figure S2 Simulation and Analysis of the General Cascade Model in Response to Two-Click Stimuli The general cascade model, equation 2 in the main text, was used with filters modeled as *l*(*t*) = sin(2*πft*)exp(−*t*/*τ*_dec_) and *q*(*t*) = exp(−*t*/*τ*_int_). The parameters were taken from the first two cells presented in detail in the main text: *f* = 14.5 kHz, *τ*_dec_ = 100 μs, and *τ*_int_ = 300 μs for Cell 1 (left column) and *f =* 5.1 kHz, *τ*_dec_ = 154 μs, and *τ*_int_ = 590 μs for Cell 2 (right column).
(A and B) Responses of tympanic vibration. *x*(*t*) denotes the signal after application of the linear filter *l*(*t*), arbitrary units, for positive second click (solid line) and negative second click (dashed line). Inter-click intervals in these two shown examples were Δ*t* = 80 μs for Cell 1 and Δ*t* = 130 μs for Cell 2.
(C and D) Corresponding responses of *J*(Δ*t*). The second click was tuned so that the maximum of *J*(Δ*t*) was equal for positive and negative second clicks. This required click amplitudes of size 1.92 and −2.49 relative to the first click for Cell 1 and 2.09 and −1.27 for Cell 2.
(E–H) Filters *L*(Δ*t*) and *Q*(Δ*t*) extracted according to equation 1 in the main text from tuning the maximum of *J*(Δ*t*) for many different values of Δ*t* (gray dots). The parameters *f, τ*_dec_, and *τ*_int_ indicated in the plots were obtained by fitting a damped harmonic oscillator and an exponential function to *L*(Δ*t*) and *Q*(Δ*t*), respectively (black lines). The initial part of *Q*(Δ*t*) shows small fluctuations that result from the oscillatory influx of charge following the tympanic vibrations. In (G), a magnified view of the initial section is shown in the inset.
(138 KB PDF).

## Abbreviation

dB SPL: decibel sound pressure level

## References

[pbio-0030008-b01] Hudspeth AJ, Logothetis NK (2000). Sensory systems. Curr Opin Neurobiol.

[pbio-0030008-b02] Robeles L, Ruggero MA (2001). Mechanics of the mammalian cochlea. Physiol Rev.

[pbio-0030008-b03] Schiolten P, Larsen ON, Michelsen A (1981). Mechanical time resolution in some insect ears. J Comp Physiol.

[pbio-0030008-b04] French AS (1988). Transduction mechanisms of mechanosensilla. Annu Rev Entomol.

[pbio-0030008-b05] Robert D, Göpfert MC (2002). Novel schemes for hearing and orientation in insects. Curr Opin Neurobiol.

[pbio-0030008-b06] Hudspeth AJ (1985). The cellular basis of hearing: The biophysics of hair cells. Science.

[pbio-0030008-b07] Hill KG (1983). The physiology of locust auditory receptors. I. Discrete depolarizations of receptor cells. J Comp Physiol A.

[pbio-0030008-b08] Gillespie PG, Walker RG (2001). Molecular basis of mechanosensory transduction. Nature.

[pbio-0030008-b09] Ricci A (2003). Active hair bundle movements and the cochlear amplifier. J Am Acad Audiol.

[pbio-0030008-b10] Evans EF, Keidel WD, Neff WD (1975). Cochlear nerve and cochlear nucleus. Auditory system. Volume 5.2, Handbook of sensory physiology.

[pbio-0030008-b11] Jameson D, Hurvich LM, editors (1972). Visual psychophysics. Volume 7.4, Handbook of sensory physiology.

[pbio-0030008-b12] Lord Rayleigh (1876). On our perception of the direction of a sound source. Nature.

[pbio-0030008-b13] Carr CE (1993). Processing of temporal information in the brain. Annu Rev Neurosci.

[pbio-0030008-b14] Grothe B, Klump GM (2000). Temporal processing in sensory systems. Curr Opin Neurobiol.

[pbio-0030008-b15] Machens CK, Schütze H, Franz A, Kolesnikova O, Stemmler MB (2003). Single auditory neurons rapidly discriminate conspecific communication signals. Nat Neurosci.

[pbio-0030008-b16] Köppl C (1997). Phase locking to high frequencies in the auditory nerve and cochlear nucleus magnocellularis of the barn owl, Tyto alba. J Neurosci.

[pbio-0030008-b17] Mason AC, Oshinsky ML, Hoy RR (2001). Hyperacute directional hearing in a microscale auditory system. Nature.

[pbio-0030008-b18] Gray EG (1960). The fine structure of the insect ear. Philos Trans R Soc London B Biol Sci.

[pbio-0030008-b19] Breckow J, Sippel M (1985). Mechanics of the transduction of sound in the tympanal organ of adults and larvae of locusts. J Comp Physiol A.

[pbio-0030008-b20] Gollisch T, Schütze H, Benda J, Herz AVM (2002). Energy integration describes sound-intensity coding in an insect auditory system. J Neurosci.

[pbio-0030008-b21] Machens CK, Stemmler MB, Prinz P, Krahe R, Ronacher B (2001). Representation of acoustic communication signals by insect auditory receptor neurons. J Neurosci.

[pbio-0030008-b22] Dallos P (1985). Response characteristics of mammalian cochlear hair cells. J Neurosci.

[pbio-0030008-b23] Garner WR (1947). The effect of frequency spectrum on temporal integration of energy in the ear. J Acoust Soc Am.

[pbio-0030008-b24] Plomp R, Bouman MA (1959). Relation between hearing threshold and duration for tone pulses. J Acoust Soc Am.

[pbio-0030008-b25] Zwislocki J (1960). Theory of temporal auditory summation. J Acoust Soc Am.

[pbio-0030008-b26] Florentine M, Fastl H, Buus S (1988). Temporal integration in normal hearing, cochlear impairment, and impairment simulated by masking. J Acoust Soc Am.

[pbio-0030008-b27] Heil P, Neubauer H (2003). A unifying basis of auditory thresholds based on temporal summation. Proc Natl Acad Sci U S A.

[pbio-0030008-b28] Lewis RS, Hudspeth AJ (1983). Voltage- and ion-dependent conductances in solitary vertebrate hair cells. Nature.

[pbio-0030008-b29] Kössl M, Boyan GS (1998). Acoustic distortion products from the ear of a grasshopper. J Acoust Soc Am.

[pbio-0030008-b30] Gollisch T, Herz AVM (2004). Input-driven components of spike-frequency adaptation can be unmasked in vivo. J Neurosci.

[pbio-0030008-b31] Gold T, Pumphrey RJ (1948). Hearing. I. The cochlea as a frequency analyzer. Proc R Soc Lond B Biol Sci.

[pbio-0030008-b32] Korenberg MJ, Hunter IW (1986). The identification of nonlinear biological systems: LNL cascade models. Biol Cybern.

[pbio-0030008-b33] van Dijk P, Wit HP, Segenhout JM (1997). Dissecting the frog inner ear with Gaussian noise. I. Application of high-order Wiener-kernel analysis. Hear Res.

[pbio-0030008-b34] Korenberg MJ, French AS, Voo SK (1988). White-noise analysis of nonlinear behavior in an insect sensory neuron: Kernel and cascade approaches. Biol Cybern.

[pbio-0030008-b35] Spekreijse H (1969). Rectification in the goldfish retina: Analysis by sinusoidal and auxiliary stimulation. Vision Res.

[pbio-0030008-b36] Marmarelis PZ, Naka K (1972). White-noise analysis of a neuron chain: An application of the Wiener theory. Science.

[pbio-0030008-b37] Benardete EA, Kaplan E (1997). The receptive field of the primate P retinal ganglion cell, II: Nonlinear dynamics. Vis Neurosci.

[pbio-0030008-b38] Emerson RC, Korenberg MJ, Citron MC (1992). Identification of complex-cell intensive nonlinearities in a cascade model of cat visual cortex. Biol Cybern.

[pbio-0030008-b39] Touryan J, Lau B, Dan Y (2002). Isolation of relevant visual features from random stimuli for cortical complex cells. J Neurosci.

[pbio-0030008-b40] Neri P (2004). Estimation of nonlinear psychophysical kernels. J Vis.

[pbio-0030008-b41] Korenberg MJ (1973). Identification of biological cascades of linear and static nonlinear systems. Proc Midwest Symp Circuit Theory.

[pbio-0030008-b42] French AS, Korenberg MJ, Järvilehto M, Kouvalainen E, Juusola M (1993). The dynamic nonlinear behavior of fly photoreceptors evoked by a wide range of light intensities. Biophys J.

[pbio-0030008-b43] Markin VS, Hudspeth AJ (1995). Gating-spring models of mechanoelectrical transduction by hair cells of the internal ear. Annu Rev Biophys Struct.

