# Supplementary material for: Disentangling Sub-Millisecond Processes within an Auditory Transduction Chain
Source: PLoS Biol. 2005 Jan 4;3(1):e8. doi: 10.1371/journal.pbio.0030008 (PMC539322; doi:10.1371/journal.pbio.0030008)
Supplement: Protocol S1 — (50 KB PDF). [file pbio.0030008.sd001.pdf]

Supporting Information for
Disentangling sub-millisecond processes within an auditory transduction chain

T. Gollisch, A.V.M. Herz

General Cascade Model

We here present a general LNLN cascade model for auditory transduction in the locust ear. After explaining the model structure, we derive its relation with the click-version of the LNLN cascade used in the main text.

The input to the general model is a time-dependent sound-pressure wave $A(t)$. The four model steps are:

1) convolution with a linear filter $l(\tau)$,

2) squaring,

3) convolution with a linear filter $q(\tau)$ yielding the effective sound intensity $J(t)$, and

4) application of a nonlinear tranformation $\tilde{g}(J)$.

The response $r(t)$ of the model is thus given by

$$r(t) = \tilde{g}\Big(J(t)\Big) \tag{S.1}$$

$$\text{with} \quad J(t) = \int_0^\infty d\tau' \; q(\tau') \cdot \left[\int_0^\infty d\tau \; l(\tau) \cdot A(t-\tau-\tau') \right]^2 . \tag{S.2}$$

As will become apparent later on, the filter functions $l(\tau)$ and $q(\tau)$ are related to, but not identical with the filters $L(\Delta t)$ and $Q(\Delta t)$ of the reduced click model. The filter functions $l(\tau)$ and $q(\tau)$ are assumed to fulfill causality conditions, i.e., $l(\tau) = q(\tau) = 0$ for $\tau < 0$. The response $r(t)$ can be interpreted as the instantaneous firing rate; in other words, $r(t) \cdot dt$ is the probability of finding a spike in the small time window dt around time t.

Note that the model does not include effects of refractoriness or adaptation, nor does it describe the measured spike-time variability, which is likely due to stochastic processes in both the generation and axonal propagation of action potentials.

We now present a self-consistent calculation, which connects the general model, Eqs. (S.1) and (S.2), to the click-model version, Eq. (1) of the main text. To start, we note that the experimental results showed that the first filter has the characteristics of a damped harmonic oscillator, whereas the second exhibits an exponential decay. We use this information to derive from the explicit formulas ($\tau > 0$)

$$l(\tau) = \sin(\omega\tau)e^{-\delta\tau}, \tag{S.3}$$

$$q(\tau) = e^{-\lambda\tau}, \tag{S.4}$$

the relationships ($\tau_1 > 0$, $\tau_2 > 0$)

$$l(\tau_1+\tau_2) = l(\tau_1)\cdot\tilde{l}(\tau_2)+l(\tau_2)\cdot\tilde{l}(\tau_1), \tag{S.5}$$

$$q(\tau_1+\tau_2) = q(\tau_1)\cdot q(\tau_2), \tag{S.6}$$

where $\tilde{l}(\tau) = \cos(\omega\tau)e^{-\delta\tau}$ denotes a phase-shifted oscillator.

In order to relate the general model to the click model, we need to investigate the responses to pairs of short, pulse-like inputs. These are mathematically modeled as Dirac delta functions at time $t_1 = 0$ and at time $t_2 = \Delta t > 0$, with amplitudes A_1 and A_2, respectively:

$$A(t) = A_1\cdot\delta(t)+A_2\cdot\delta(t-\Delta t). \tag{S.7}$$

The resulting effective sound intensity $J(t)$ is a function of time. Considering our experimental approach, we are interested in the total (integrated) probability that a spike occurs. This probability is given by

$$p = 1-\exp\left[-\int_0^\infty dt\ \tilde{g}\Big(J(t)\Big)\right]. \tag{S.8}$$

We cannot calculate this integral explicitly without making specific assumptions about the function $\tilde{g}(\,\cdot\,)$. However, we can expect that $J(t)$ has a rather stereotypic, sharply peaked form if click

stimuli are used that are adjusted to yield the same maximum J. This is supported by simulation results that indicate that for our experimental paradigm, both the overall time evolution of $J(t)$ as well as the time T after the second click when the maximum of $J(t)$ is reached are almost identical for the different click combinations (see Fig. S2, C and D).

In addition, $\tilde{g}(\cdot)$ will typically be a monotonically increasing function. We may thus assume that the integral in Eq. (S.8) is dominated by the maximum J of $J(t)$ and that this maximum is obtained at approximately the same time T after the second click.

Before we calculate the required $J(\Delta t+T)$, let us note that the assumption of a single instant of spike generation at time $\Delta t+T$ can be relaxed by using the fact that the values of $\tilde{g}(J(t))$ in some small window $[T_1,T_2]$ around $\Delta t+T$ will have the largest influence on spike generation. Linearizing the function $\tilde{g}(J)$ then suggests that the integral

$$\int_{T_1}^{T_2} dt \;\; J(t) \tag{S.9}$$

is the relevant quantity for calculating the spike probability. In the following, we will only present calculations for the case where $J(t)$ is considered at a fixed time $\Delta t+T$, but the more general case, Eq. (S.9), leads to the same conclusions; every integral in the following calculation just has to be supplemented with an additional integral over the interval $[T_1,T_2]$.

To calculate $J \equiv J(\Delta t+T)$, we substitute $A(t)$ from Eq. (S.7) into Eq. (S.2). We can solve the inner integral directly because of the delta functions and obtain after evaluating the expression at $t=\Delta t+T$

$$J = \int_0^\infty d\tau \;\; \Big[A_1 \cdot l(\Delta t+T-\tau) + A_2 \cdot l(T-\tau)\Big]^2 \cdot q(\tau). \tag{S.10}$$

Splitting up the integral together with applying the causality condition yields

$$\begin{aligned} J &= \int_T^{\Delta t+T} d\tau \;\; {A_1}^2 \cdot [l(\Delta t+T-\tau)]^2 \cdot q(\tau) \\ &\quad + \int_0^T d\tau \;\; \Big[A_1 \cdot l(\Delta t+T-\tau) + A_2 \cdot l(T-\tau)\Big]^2 \cdot q(\tau). && \text{(S.11)} \\ &= \int_0^{\Delta t} d\tau \;\; {A_1}^2 \cdot [l(\tau)]^2 \cdot q(\Delta t+T-\tau) \\ &\quad + \int_0^T d\tau \;\; \Big[A_1 \cdot l(\Delta t+\tau) + A_2 \cdot l(\tau)\Big]^2 \cdot q(T-\tau). && \text{(S.12)} \end{aligned}$$

The last step was obtained by variable transformations. We now apply the formulas Eqs. (S.5) and (S.6) to isolate the Δt-dependences and find

$$\begin{aligned} J &= \int_0^{\Delta t} d\tau \; {A_1}^2 \cdot [l(\tau)]^2 \cdot q(\Delta t) \cdot q(T-\tau) \\ &\quad + \int_0^{T} d\tau \; \left[A_1 \cdot l(\Delta t) \cdot \tilde{l}(\tau) + A_1 \cdot l(\tau) \cdot \tilde{l}(\Delta t) + A_2 \cdot l(\tau)\right]^2 \cdot q(T-\tau). \end{aligned} \tag{S.13}$$

By multiplying out the square in the second integral, we finally see that the effective sound intensity decomposes into four terms:

$$\begin{aligned} J &= {A_1}^2 \cdot q(\Delta t) \cdot \int_0^{\Delta t} d\tau \; [l(\tau)]^2 \cdot q(T-\tau) \\ &\quad + \left[A_1 \cdot \tilde{l}(\Delta t) + A_2\right]^2 \cdot \int_0^{T} d\tau \; [l(\tau)]^2 \cdot q(T-\tau) \\ &\quad + {A_1}^2 \cdot l^2(\Delta t) \cdot \int_0^{T} d\tau \; [\tilde{l}(\tau)]^2 \cdot q(T-\tau) \\ &\quad + 2 \cdot A_1 \cdot l(\Delta t) \cdot \left[A_1 \cdot \tilde{l}(\Delta t) + A_2\right] \cdot \int_0^{T} d\tau \; \tilde{l}(\tau) \cdot l(\tau) \cdot q(T-\tau). \end{aligned} \tag{S.14}$$

The last term contains an integral over a product of a sine (from $l(t)$) and a cosine (from $\tilde{l}(t)$) and will thus be small compared to the other integrals, as positive and negative contributions nearly cancel each other out. We therefore neglect this term. Using the definitions

$$k = \int_0^{T} d\tau \; [l(\tau)]^2 \cdot q(T-\tau), \tag{S.15}$$

$$\zeta = \frac{1}{k} \cdot \int_0^{T} d\tau \; [\tilde{l}(\tau)]^2 \cdot q(T-\tau), \tag{S.16}$$

$$\gamma(\Delta t) = \frac{1}{k} \cdot \int_0^{\Delta t} d\tau \; [l(\tau)]^2 \cdot q(T-\tau), \tag{S.17}$$

Eq. (S.14) can be written as

$$\begin{aligned} J &= {A_1}^2 \cdot q(\Delta t) \cdot \gamma(\Delta t) \cdot k + \left[A_1 \cdot \tilde{l}(\Delta t) + A_2\right]^2 \cdot k + {A_1}^2 \cdot [l(\Delta t)]^2 \cdot \zeta \cdot k \\ &= {A_1}^2 \cdot \left\{q(\Delta t) \cdot \gamma(\Delta t) + [l(\Delta t)]^2 \cdot \zeta\right\} \cdot k + \left[A_1 \cdot \tilde{l}(\Delta t) + A_2\right]^2 \cdot k. \end{aligned} \tag{S.18}$$

Absorbing the constant factor k in the functional relation between J and p, we obtain the same functional dependence of J on A_1 and A_2 as in the click model, Eq. (1) in the main text. By

comparison, we find

$$L(\Delta t) = \tilde{l}(\Delta t), \tag{S.19}$$

$$Q(\Delta t) = q(\Delta t) \cdot \gamma(\Delta t) + [l(\Delta t)]^2 \cdot \zeta. \tag{S.20}$$

$L(\Delta t)$ is thus a phase-shifted version of the tympanum's response function $l(\Delta t)$, but retains the important resonance characteristics such as natural frequency and decay constant. In fact, as the oscillation of $l(\Delta t)$ is found to be significantly faster than its decay ($\omega = 2\pi f > \delta = 1/\tau_{\text{dec}}$, cf. Fig. 5), $\tilde{l}(\Delta t)$ is approximately proportional to the derivative of $l(\Delta t)$. Hence, $L(\Delta t)$ measures the velocity of the tympanic vibration. Note that this interpretation is consistent with the initial jump of $L(\Delta t)$ at $t = 0$, which is not expected for the displacement of an impulse-driven oscillator, but rather for its velocity.

$Q(\Delta t)$ captures the response function of electrical integration given by $q(\Delta t)$ up to small corrections. The correction factor ζ results from the ratio between two integrals, which differ in that they contain $[l(\tau)]^2$ and $[\tilde{l}(\tau)]^2$, respectively. As the phase of these two functions does not contribute strongly to the integral, ζ is a constant near unity. For large enough Δt, the term $[l(\Delta t)]^2 \cdot \zeta$ is negligible, as it is quadratic in $l(\Delta t)$, which itself goes to zero. For small Δt, though, this term may lead to a small oscillatory contribution to $Q(\Delta t)$. The biophysical mechanism giving rise to these fluctuations is the oscillatory influx of charge, which results from the vibration of the tympanum. Its effect can also be seen in the filter $Q(\Delta t)$ extracted from simulations of the general cascade model, Fig. S2, G and H. In our experiments, however, the recording time was not sufficient to detect these fluctuations with high enough accuracy.

The factor $\gamma(\Delta t)$ in Eq. (S.20) approaches unity also due to the fast decay of $[l(\Delta t)]^2$, which yields the integral in Eq. (S.17) equal fo k, Eq. (S.15), for large enough Δt. Together with the vanishing $[l(\Delta t)]^2 \cdot \zeta$ term, we find that $Q(\Delta t) \approx q(\Delta t)$ if Δt is large enough. From the time scale of $L(\Delta t)$, Fig. 5, this corresponds to $\Delta t \gtrsim 100$ or 200 µs. For small Δt, on the other hand, the factor $\gamma(\Delta t)$ approaches zero and suppresses the contribution from $q(\Delta t)$ leading to an initial rising phase of $Q(\Delta t)$ in contrast to the sharp onset of $q(\Delta t)$. This effect is seen in the data (Fig. 5) as well as expected from the notion that, for short Δt, the inter-click interval is too short

to allow for a transduction current induced by the first click alone.

The above derivation is based on two main assumptions: 1) that Eqs. (S.5) and (S.6) apply to $l(\tau)$ and $q(\tau)$ and 2) that the maximum value of the effective sound intensity $J(t)$ is obtained at about the same time after the second click.

The first assumption rests on the experimentally extracted forms of $L(\Delta t)$ and $Q(\Delta t)$. The derivation is thus not deductive, but instead leads to a self-consistent interpretation of the relations between l, q, L, and Q. The derivation explicitly uses the oscillator characteristics of $l(\tau)$ and the leaky-integrator properties of $q(\tau)$. For other experimental systems, the relations between l, q, L, and Q may be different. This would not at all preclude an experimental examination of the filters based on the same approach with click stimuli, but it may alter the interpretation of the extracted filters with respect to the corresponding general cascade model.

The second assumption is motivated by the expected sharp deflections of the transduction currents in response to click stimuli. Numerical results support this view. In Fig. S2, simulations of the cascade model, Eq. (S.2), are shown for two sets of parameters, which were taken from the first two experimental examples presented in the main text, Fig. 5. The second click of two-click stimuli was tuned so that the maximum of $J(t)$ obtained a fixed value. Whereas the signal $x(t)$ after the application of the first linear filter $l(\tau)$ clearly differs if the second click is presented in the positive or negative direction (panels A and B), $J(t)$ is almost identical in both cases (panels C and D). Even though the time of the maximum of $J(t)$ does not exactly coincide, adjusting the maximum to a predefined value can be used to accurately extract the filters $L(\Delta t)$ and $Q(\Delta t)$. This is shown in panels E–H, where the filters of the two parameter sets were extracted in the same way as in the main text, Eq. (1). Comparison with Fig. 5 demonstrates the consistency of the data with the full cascade model, Eq. (S.2).
