# Supplementary material for: Disentangling Sub-Millisecond Processes within an Auditory Transduction Chain
Source: PLoS Biol. 2005 Jan 4;3(1):e8. doi: 10.1371/journal.pbio.0030008 (PMC539322; doi:10.1371/journal.pbio.0030008)
Supplement: Figure S1 — The four panels show different examples of stimuli used in our study. Each panel illustrates the computer-generated pulse signal that drives the loud speaker (upper trace) and the resulting air-pressure fluctuations as measured with a high-precision microphone at the site of the animal's ear (lower trace). The computer-generated clicks are triangular with a total width of 20 μs. The stimuli shown are (A) a single click, (B) a double click with a peak-to-peak interval Δt = 50 μs, (C) a double click with Δt = 500 μs, and (D) another double click with Δt = 500 μs whose second click points in the oppositve (“negative”) direction. The measurements of air-pressure fluctuations indicate a slight broadening of the click width and some residual vibrations, but they nevertheless present a good approximation of the sharp original pulses. (10 KB PDF). [file pbio.0030008.sg001.pdf]

Figure S1

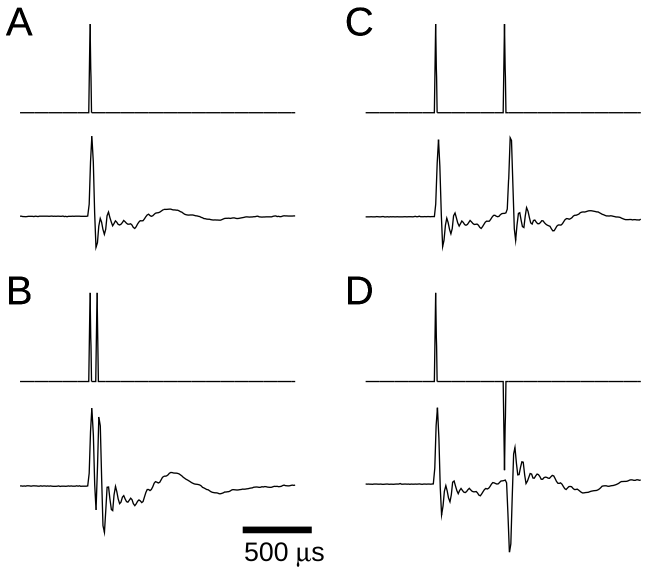

Examples of click stimuli.
The four panels show different examples of stimuli used in our study. Each panel illustrates the computer-generated pulse signal that drives the loud speaker (upper trace) and the resulting air-pressure fluctuations as measured with a high-precision microphone at the site of the animal's ear (lower trace). The computer-generated clicks are triangular with a total width of 20 µs. The stimuli shown are (**A**) a single click, (**B**) a double click with a peak-to-peak interval $\Delta t = 50\,\mu s$, (**C**) a double click with $\Delta t = 500\,\mu s$, and (**D**) another double click with $\Delta t = 500\,\mu s$ whose second click points in the oppositve ("negative") direction. The measurements of air-pressure fluctuations indicate a slight broadening of the click width and some residual vibrations, but they nevertheless present a good approximation of the sharp original pulses.
