# Supplementary material for: Disentangling Sub-Millisecond Processes within an Auditory Transduction Chain
Source: PLoS Biol. 2005 Jan 4;3(1):e8. doi: 10.1371/journal.pbio.0030008 (PMC539322; doi:10.1371/journal.pbio.0030008)
Supplement: Figure S2 — The general cascade model, equation 2 in the main text, was used with filters modeled as l(t) = sin(2πft)exp(−t/τ dec) and q(t) = exp(−t/τ int). The parameters were taken from the first two cells presented in detail in the main text: f = 14.5 kHz, τ dec = 100 μs, and τ int = 300 μs for Cell 1 (left column) and f = 5.1 kHz, τ dec = 154 μs, and τ int = 590 μs for Cell 2 (right column). (A and B) Responses of tympanic vibration. x(t) denotes the signal after application of the linear filter l(t), arbitrary units, for positive second click (solid line) and negative second click (dashed line). Inter-click intervals in these two shown examples were Δt = 80 μs for Cell 1 and Δt = 130 μs for Cell 2. (C and D) Corresponding responses of J(Δt). The second click was tuned so that the maximum of J(Δt) was equal for positive and negative second clicks. This required click amplitudes of size 1.92 and −2.49 relative to the first click for Cell 1 and 2.09 and −1.27 for Cell 2. (E–H) Filters L(Δt) and Q(Δt) extracted according to equation 1 in the main text from tuning the maximum of J(Δt) for many different values of Δt (gray dots). The parameters f, τ dec, and τ int indicated in the plots were obtained by fitting a damped harmonic oscillator and an exponential function to L(Δt) and Q(Δt), respectively (black lines). The initial part of Q(Δt) shows small fluctuations that result from the oscillatory influx of charge following the tympanic vibrations. In (G), a magnified view of the initial section is shown in the inset. (138 KB PDF). [file pbio.0030008.sg002.pdf]

Figure S2

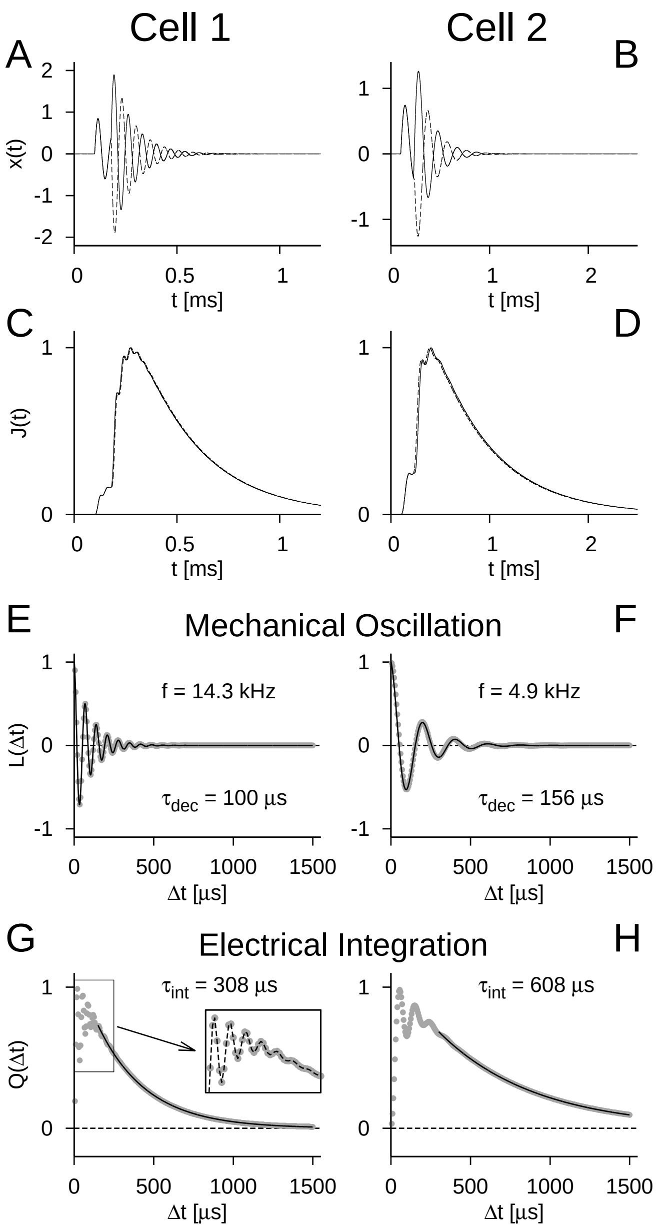

Simulation and analysis of the general cascade model in response to two-click stimuli.

The general cascade model, Eq. (2) in the main text, was used with filters modeled as $l(t) = \sin(2\pi f t)\exp(-t/\tau_{\mathrm{dec}})$ and $q(t) = \exp(-t/\tau_{\mathrm{int}})$. The parameters were taken from the first two cells presented in detail in the main text: $f = 14.5$ kHz, $\tau_{\mathrm{dec}} = 100$ µs, and $\tau_{\mathrm{int}} = 300$ µs for Cell 1 (left column) and $f = 5.1$ kHz, $\tau_{\mathrm{dec}} = 154$ µs, and $\tau_{\mathrm{int}} = 590$ µs for Cell 2 (right column). (**A**,**B**) Responses of tympanic vibration. $x(t)$ denotes the signal after application of the linear filter $l(\tau)$, arbitrary units, for positive second click (solid line) and negative second click (dashed line). Inter-click intervals in these two shown examples were $\Delta t = 80$ µs for Cell 1 and $\Delta t = 130$ µs for Cell 2. (**C**,**D**) Corresponding responses of $J(t)$. The second click was tuned so that the maximum of $J(t)$ was equal for positive and negative second clicks. This required click amplitudes of size 1.92 and -2.49 relative to the first click for Cell 1 and 2.09 and -1.27 for Cell 2. (**E–H**) Filters $L(\Delta t)$ and $Q(\Delta t)$ extracted according to Eq. (1) in the main text from tuning the maximum of $J(t)$ for many different values of Δt (gray dots). The parameters f, τ_{dec}, and τ_{int} indicated in the plots were obtained by fitting a damped harmonic oscillator and an exponential function to $L(\Delta t)$ and $Q(\Delta t)$, respectively (black lines). The initial part of $Q(\Delta t)$ shows small fluctuations that result from the oscillatory influx of charge following the tympanic vibrations. In panel G, a magnified view of the initial section is shown in the inset.
